# ﻿Mitochondrial phylogenomics of pygmy grasshoppers (Orthoptera, Tetrigidae, Metrodorinae): descriptions of a new genus, two new species, and new synonyms from China

**DOI:** 10.3897/zookeys.1236.145914

**Published:** 2025-05-05

**Authors:** Yuemei Li, Shixiong Leng, Jiasong He, Weian Deng, Delong Guan

**Affiliations:** 1 Key Laboratory of Ecology of Rare and Endangered Species and Environmental Protection, Guangxi Normal University, Ministry of Education, Guilin 541006, China Guangxi Normal University Guilin China; 2 Guangxi Key Laboratory of Rare and Endangered Animal Ecology, Guangxi Normal University, Guilin 541006, China Guangxi Daguishan Crocodile Lizard National Nature Reserve Hezhou China; 3 College of Life Sciences, Guangxi Normal University, Guilin 541006, China Hechi University Hechi China; 4 Guangxi Daguishan Crocodile Lizard National Nature Reserve, Hezhou, Guangxi 542824, China Guangxi Normal University Guilin China; 5 Guangxi Key Laboratory of Sericulture ecology and Applied intelligent technology, Hechi University, Hechi, China Guangxi Daguishan Crocodile Lizard National Nature Reserve Hezhou China; 6 School of Chemistry and Bioengineering, Hechi University, Yizhou, Guangxi 546300, China Hechi University Hechi China

**Keywords:** *Macromotettixoides*, Metrodoridae, mitochondrial genome, new taxa, ovipositor, phylogeny, taxonomy

## Abstract

The Chinese wingless brachypronotal pygmy grasshoppers of the subfamily Metrodorinae have often been classified within the genus *Macromotettixoides*. In this study, two undescribed species of wingless brachypronotal pygmy grasshoppers belonging to Metrodorinae were collected. To elucidate their taxonomic positions, the complete mitochondrial genomes of these two species were sequenced and analyzed. Phylogenetic analyses were conducted using 13 protein-coding genes (PCGs) from 28 tetrigid mitogenomes. Genetic distances and divergence times were estimated. Our results indicate that one species represents a new genus within Metrodorinae, while the other is a new species of *Macromotettixoides*. Consequently, a new genus and two new species of Metrodolrinae from China are described and illustrated: *Edentatettix* Deng, **gen. nov.** containing *Edentatettixleyeensis* Deng, **sp. nov.**, and *Macromotettixoidesyaana* Deng, **sp. nov.** Additionally, five new synonyms are proposed: *Hainantettixangustivertex* (Zha & Peng, 2021), **syn. nov.** and *Macromotettixoidesangustivertex* Zha & Peng, 2021, **syn. nov.** of *Hainantettixstrictivertex* Deng, 2020; *Hyboellabadagongshanensis Zheng*, 2013, **syn. nov.**, *Macromotettixoidesbadagongshanensis* (Zheng, 2013), **syn. nov.**, and *Macromotettixoideswuyishana* Zheng, 2013, **syn. nov.** of *Macromotettixoidesjiuwanshanensis* Zheng, Wei & Jiang, 2005. For the first time, edentate ovipositors constituting an important taxonomic character within Tetrigidae is reported and discussed.

## ﻿Introduction

In the highly diverse orthopteran insects, tetrigids (Orthoptera: Tetrigidae) represent a relatively ancient group of orthopteran insects. Among them, Metrodorinae Bolívar, [1887] is one of the three largest subfamilies in Tetrigidae and currently includes 105 genera containing more than 648 species distributed around the world (Deng 2016; Cigliano et al. 2024), with 16 genera (including the new genus *Edentatettix* Deng, gen. nov.) found in China. Although Metrodorinae has a wide distribution in the world, there are still many species with classification disputes due to the similarity and diversity of morphological characteristics. Since the establishment of Metrodorinae, numerous species within it have undergone transfers between genera, and the genera within the subfamily have been revised frequently (Storozhenko 2014; Tumbrinck and Telnov 2014; Tumbrinck 2019; Skejo et al. 2019; Peng et al. 2021; Kasalo 2022; Kasalo et al. 2023b).

Pygmy grasshoppers (Tetrigidae) is a taxonomic challenging group, exhibiting striking polymorphism in various morphological traits such as body color, patterns, wing lengths, and pronotum size and shape. Long et al. (2023) systematically scrutinized type specimens of pygmy grasshoppers stored in China, revealing 23 new synonyms of *Tetrixjaponica* (Bolívar, 1887), indicating notable morphological diversity. Concurrently, Zhang et al. (2023) found similar variability in *Tetrixjaponica* morphology throughout their life cycles. Given the complexity and variability of morphological features within Tetrigidae, relying solely on these traits often leads to recurrent classification errors, hindering the achievement of the rigorous accuracy standards required in contemporary species classification. Consequently, it is necessary to utilize molecular data as an auxiliary verification tool to ensure the accuracy of classification. In recent years, with the rapid development of high-throughput sequencing, especially the progress of whole mitochondrial genome sequencing, insect mitochondrial genomes have been widely used as a molecular marker to investigate phylogenetic relationships, biological identification, and the genetic structure of populations (Boore 1999; Cameron 2014; Song et al. 2019; Nie et al. 2021; Li et al. 2021b). Mitogenomes have greatly improved our understanding of the phylogenetics of Tetrigidae, and some scholars have begun to use these methods to answer questions about the taxonomy and phylogeny of tetrigids (Fang et al. 2010; Pavón-Gozalo et al. 2012; Lin et al. 2015; Chen et al. 2018; Adžić et al. 2020; Huang et al. 2022; Kasalo et al. 2023a).

In this study, we obtained two unknown species of wingless pygmy grasshoppers of Metrodorinae. To determine their taxonomic positions, we sequenced their mitochondrial genomes, constructed molecular phylogenetic relationships, and estimated genetic distances and divergence times. Finally, it was determined that one of them belongs to a new genus of Metrodorinae, while the other belongs to a new species of the genus *Macromotettixoides*. We establish a new genus *Edentatettix* Deng, gen. nov., characterized by the absence or degeneration of the unique saw-like teeth in the female ovipositor. *Edentatettixleyeensis* Deng, sp. nov. is described as type species. Meanwhile, *Macromotettixoidesyaana* Deng, sp. nov., is described as new to science. Based on a re-examination of type specimens, we propose five critical taxonomic revisions as follows: 1) synonymization of *Hainantettixstrictivertex* Deng, 2020 with *H.angustivertex* (Zha & Peng, 2021); 2) reclassification of *Macromotettixoidesangustivertex* Zha & Peng, 2021 under *Hainantettix*; 3) consolidation of *Macromotettixoidesjiuwanshanensis* Zheng, Wei & Jiang, 2005 with *Hyboellabadagongshanensis* Zheng, 2013, *M.badagongshanensis* (Zheng, 2013), and *M.wuyishana* Zheng, 2013 based on overlapping diagnostic characters. In addition, the taxonomic significance of toothless ovipositors in Tetrigidae is discussed.

## ﻿Materials and methods

### ﻿Mitogenome sequencing, assembly, annotation and analysis

Total genomic DNA was extracted from muscle tissues of the hind femur using TIANamp Genomic DNA Kit (TIANGEN) and sent to Berry Genomics (Beijing, China) for genomic sequencing using Next Generation Sequencing (NGS) method. Separate 350-bp insert libraries were created from the whole genomic DNA and sequenced using the Illumina HiSeq X Ten sequencing platform. A total of 5 Gb of 150-bp paired-end (PE) reads were generated in total for each sample.

The mitochondrial genome was assembled by NOVOPlasty 4.2.1 and annotated using the MITOS2 Web Server (http://mitos2.bioinf.uni-leipzig.de/index.py, accessed on 17 May 2024; Donath et al. 2019). The annotated mitogenome sequences were checked in CLC Genomics Workbench 12.0.1, MEGA 11.0.13, and Geneious Prime 11.0.18. The maps of the mitogenomes were generated using the Proksee website (https://proksee.ca, accessed on 10 August 2024, Grant et al. 2023). The nucleotide composition of the mitogenome, PCGs, three codon positions of PCGs, tRNA genes, rRNA genes, and the control regions (CR) was computed in PhyloSuite v.1.2.3 and MEGA 11.0.13. The AT and GC skews were calculated using the formula: AT-skew = (A-T) / (A + T) and GC-skew = (G-C) / (G + C).

### ﻿Phylogenetic analysis

To determine the phylogenetic positions of two new species in Metrodoridae, a total of 30 mitogenomes, including 28 downloaded from NCBI and two from this study were employed. *Mirhipipteryxandensis* (Ripipterygidae: Ripipteryginae) and *Ellipesminuta* (Tridactylidae: Tridactylinae) were selected as outgroups (Table 1). The analysis was performed using PhyloSuite v. 1.2.3. Redundant sequences were removed, and PCGs in the mitochondrial genome were extracted, aligned in batches, and concatenated with MAFFT (Katoh and Standley 2013). Thirteen PCGs were aligned and concatentated through the MAFFT v. 7.505 (Katoh and Standley 2013) plugin in PhyloSuite v. 1.2.3. ModelFinder v. 2.2.0 (Kalyaanamoorthy et al. 2017) was used to select the best-fit model using AICc and BICc standards. The phylogenetic tree was assessed with the maximum likelihood method and the bayesian inference method. The maximum likelihood estimations with IQ-TREE v. 2.2.0 (Nguyen et al. 2015) under the Edge-linked partition model with 15,000 ultrafast bootstrap replicates (Minh et al. 2013) and Bayesian inference used MrBayes v. 3.2.7a under the GTR+F+I+G4 model (with two parallel runs, 2,000,000 generations). Genetic distances were calculated using the Tajima-Nei model based on the sequence of 13 PCGs with the bootstrap method of 1000 replications using MEGA 11.0.13.

**Table 1.** Accession numbers and references of the mitogenomes of Tetrigidae included in this study.

Subfamily	Species	Accession number	Reference
Batrachideinae	*Saussurellaborneensis*	MZ169555	Deng et al. 2021
Tripetalocerinae	*Tripetaloceroidestonkinensis*	MW770353	Zhang et al. 2021
Scelimeninae	*Eucriotettixoculatus*	MT162546	Li et al. 2021a
	*Loxilobusprominenoculus*	MT162545	Li et al. 2021b
Metrodorinae	*Bolivaritettixsikkinensis*	KY123120	Yang et al. 2017b
	*Bolivaritettixyuanbaoshanensis*	KY123121	Yang et al. 2017b
	*Bolivaritettixlativertex*	MN083173	Chang et al. 2020
	*Macromotettixoidesmaoershanensis*	OR030790	Luo et al. 2024
	*Macromotettixoidesbrachycorna*	OR003899	Luo et al. 2024
	*Macromotettixoidesorthomargina*	OR030789	Luo et al. 2024
	*Macromotettixoidesyaana* Deng, sp. nov.	PQ826485	This study
	*Edentatettixleyeensis* Deng, sp. nov.	PQ826484	This study
	*Systolederusspicupennis*	MH791445	Yang 2017a
	*Teredorusbashanensis = Systolederusbashanensis* (Devriese & Husemann, 2023)	MZ041208	Li et al. 2021c
	*Teredorusanhuiensis = Systolederusanhuiensis* (Devriese & Husemann, 2023)	NC_071822	Unpublished
	*Teredorusguangxiensis = Systolederuszhengi* (Devriese & Husemann, 2023)	NC_082935	Guan et al. 2024
	*Teredorus hainanensis = Systolederushainanensis* (Devriese & Husemann, 2023)	NC_063117	Li et al. 2021c
	*Teredorusnigropennis = Systolederusnigropennis* (Devriese & Husemann, 2023)	MN938922	Li et al. 2020b
Tetriginae	*Coptotettixlongtanensis*	OK540319	Unpublished
	*Coptotettixlongjiangensis*	KY798413	Lin et al. 2017
	*Euparatettixtridentatus*	NC_082933	Guan et al. 2024
	*Euparatettixvariabilis*	NC_046542	Chang et al. 2020
	*Euparatettixbimaculatus*	NC_046541	Chang et al. 2020
	*Exothotettixguangxiensis*	NC_082934	Guan et al. 2024
	*Formosatettixqinlingensis*	KY798412	Lin et al. 2017
	*Alulatettixyunnanensis*	NC_018542	Xiao et al. 2012a
	*Tetrixjaponica*	NC_018543	Xiao et al. 2012b
	*Tetrixruyuanensis = Tetrixjaponica* (Long et al., 2023)	NC_046412	Chang et al. 2020
Outgroup	*Mirhipipteryxandensis*	NC_028065	Song et al. 2015
	*Ellipesminuta*	NC_014488	Sheffield et al. 2010

### ﻿Divergence time analysis

The divergence times were estimated using BEAST v. 1.8.4 (Drummond and Rambaut 2007; Drummond et al. 2012), and the calibration times estimated from previous studies were queried from the timetree.org website (http://timetree.org/, accessed on 9 December 2024; Kumar et al. 2022). The nodes of *Tetrix* and *Alulatettix* (4.35~7.98 MYA) was selected for calibration (Song et al. 2015). The parameters, including time priors and prior distributions, were set as “Yule Process” and “Normal”. The range was strictly set according to the queried calibration times. The uncorrelated relaxed clock model was selected with the relaxed distribution set as lognormal, and the same partition generated from the IQ-TREE was adopted. Finally, the MCMC generations and burn-ins were set as 10 million and 10%, respectively. The generated trees were imported into the Treeannotator to yield a consensus tree. The resulting phylogenetic tree was viewed and edited in ChiPlot (v. 2.6.1) (https://www.chiplot.online/, accessed on 10 December 2024; Xie et al. 2023).

### ﻿Taxonomy, nomenclature, terminology, and measurements

Taxonomy follows the Orthoptera Species File [OSF] (Cigliano et al. 2024), a database of Orthoptera taxonomy. Nomenclature follows the International Code of the Zoological Nomenclature (ICZN 1999). Morphological terminology and landmark-based measurement methods followed those used by Zheng (2005a), Deng et al. (2007), Tumbrinck and Telnov (2014), Tumbrinck (2019), Tan and Artchawakom (2015), Muhammad et al. (2018), and Zha et al. (2017). Measurements are given in millimeters (mm). Grasshopper specimens were examined using a Motic-SMZ-168 stereo-microscope and photographed using a KEYENCE VHX-600 Digital Microscope (Keyence Corporation, Osaka, Japan). All images were processed with Adobe Photoshop 24.0.0. The distribution map was prepared by ArcMap 10.8.1 and edited in Adobe Photoshop 24.0.0.

The species of *E.leyeensis* Deng, sp. nov. was collected from Wutaishan Forest Park, Leye County, Guangxi Province, China (24°51'11"N, 106°32'17"E) on 23 August 2021 by Wei-An Deng. Specimens of *M.yaana* Deng, sp. nov. were collected from Longdong (Ganyanggou), Baoxing County, Yaan City, Sichuan Province, China (30°24′19″N, 102°35′45″E) on 2 August 2016 by Wei-An Deng. The collected specimens were preserved in 100% anhydrous ethanol and stored in the refrigerator at –20 °C at the College of Life Science, Guangxi Normal University, Guilin, China (CLSGNU).

### ﻿Type specimen depositories

The specimens examined in this study, including all holotypes and paratypes, have been deposited in the following institutions:

**CLSGNU**College of Life Science, Guangxi Normal University, Guilin, China;

**EMHU** Entomological Museum of Hechi University, Hechi, China;

**IZSNU**Institute of Zoology, Shaanxi Normal University, Xi’an, China;

**HNU** Huaibei Normal University, Huaibei, Anhui, China.

## ﻿Results

### ﻿Mitogenome characteristics of *E.leyeensis* Deng, sp. nov. and *M.yaana* Deng, sp. nov.

Genome organization and nucleotide composition

**Figure 1.**
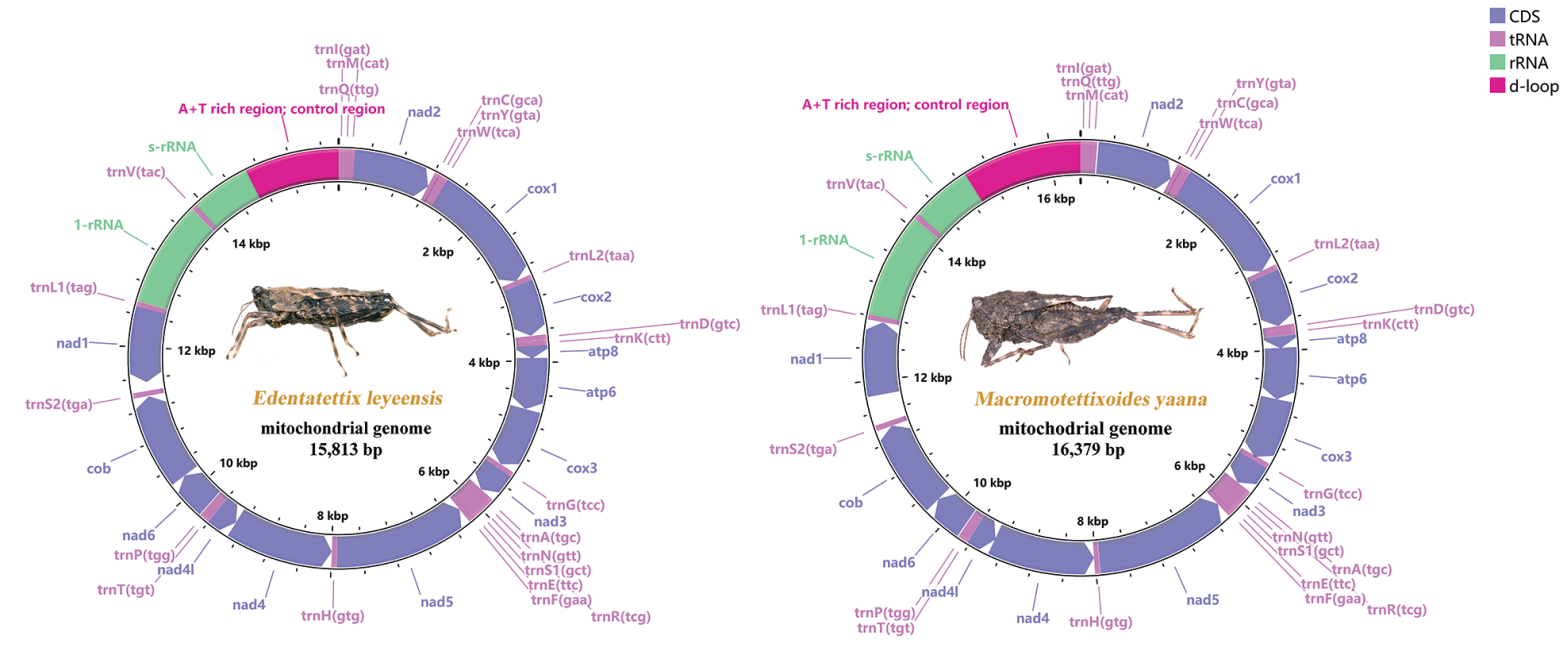
Circular mitochondrial maps of *E.leyeensis* Deng, sp. nov. and *M.yaana* Deng, sp. nov.

The mitogenomes of *E.leyeensis* Deng, sp. nov. and *M.yaana* Deng, sp. nov. are circularized, with sizes of 15,813 bp and 16,379 bp, respectively (Fig. 1). Both mitogenomes contain 13 PCGs, 22 tRNAs, 2 rRNA (*rrnS* and *rrnL*), and a control region. Most genes (9 PCGs and 14 tRNA genes) were encoded on the majority strand (J-strand), and the rest of the genes (4 PCGs, 8 tRNAs, and 2 rRNAs) were located on the minority strand (N-strand) (Table 2).

**Table 2.** Mitochondrial genome organization of *E.leyeensis* Deng, sp. nov. and *M.yaana* Deng, sp. nov.

Genes	Strand	Anticodon	Location	Length (bp)	Intergenic nucleotides	Start codon	Stop codon
*trnI*	J	GAT	1–64/1–65	64/65	0/.	–	–
*trnQ*	N	TTG	66–134/.	69/.	1/.	–	–
*trnM*	J	CAT	134–201/134–205	68/72	-1/.	–	–
*nad2*	J	–	202–1209/221–1216	1008/996	0/15	ATG/ATT	TAA/.
*trnW*	J	TCA	1208–1272/1215–1282	65/68	-2/.	–	–
*trnC*	N	GCA	1265–1326/1275–1338	62/64	-8/.	–	–
*trnY*	N	GTA	1327–1389/1339–1402	63/64	0/.	–	–
*cox1*	J	–	1387–2925/1400–2938	1539/.	-3/.	ATC/.	TAA/.
*trnL2*	J	TAA	2921–2983/2934–2997	63/64	0/-5	–	–
*cox2*	J	–	2985–3662/2999–3682	678/684	1/.	ATG/.	TAA/.
*trnD*	J	GTC	3666–3728/3682–3744	63/.	3/-1	–	–
*trnK*	J	CTT	3732–3797/3746–3811	67/66	2/1	–	–
*atp8*	J	–	3797–3958/3815–3973	162/159	-1/3	ATA/ATG	TAA/.
*atp6*	J	–	3952–4623/3967–4638	672/.	-7/.	ATG/.	TAA/.
*cox3*	J	–	4623–5411/4638–5444	789/807	-1/.	ATG/.	TAA/.
*trnG*	J	TCC	5411–5473/5428–5489	63/62	-1/-17	–	–
*nad3*	J	–	5474–5827/5490–5843	354/.	0/.	ATT/.	TAG/.
*trnA*	J	TGC	5826–5892/5842–5906	67/65	-2/.	–	–
*trnR*	J	TCG	5892–5953/5906–5965	62/60	-1/.	–	–
*trnN*	J	GTT	5954–6017/5966–6032	64/67	0/.	–	–
*trnS1*	J	GCT	6018–6081/6033–6099	64/67	0/.	–	–
*trnE*	J	TTC	6082–6145/6100–6163	64/.	0/.	–	–
*trnF*	N	GAA	6144–6207/6162–6223	64/62	-2/.	–	–
*nad5*	N	–	6208–7930/6224–7946	1723/.	0/.	ATG/.	T(AA)/.
*trnH*	N	GTG	7933–7996/7951–8015	64/65	2/4	–	–
*nad4*	N	–	7996–9315/8015–9340	1320/1326	-1/.	ATA/ATG	TAG/.
*nad4l*	N	–	9315–9602/9334–9624	288/291	-1/-7	ATT/.	TAA/.
*trnT*	J	TGT	9605–9666/9627–9688	62/.	2/.	–	–
*trnP*	N	TGG	9667–9729/9689–9755	63/67	0/.	–	–
*nad6*	J	–	9740–10225/9769–10248	486/480	10/13	ATT/ATA	TAA/.
*cob*	J	–	10225–11361/10248–11387	1137/1140	-1/.	ATG/.	TAA/.
*trnS2*	J	TGA	11370–11434/11386–11452	65/67	8/-2	–	–
*nad1*	N	–	11450–12491/11811–12749	942/945	115/358	ATT/.	TAA/.
*trnL1*	N	TAG	12492–12555/12750–12813	64/.	0/.	–	–
*rrnL*	N	–	12560–13852/12815–14099	1293/1285	4/1	–	–
*trnV*	N	TAC	13857–13924/14103–14170	68/.	4/3	–	–
*rrnS*	N	–	13923–14657/14170–14913	735/744	-2/-1	–	–
CR	–	–	14658–15813/14914–16379	1156/1466	–	–	–

Note: “H” indicates the majority strand and “L” indicates the minority strand.

The AT-skew and nucleotide composition of the mitogenomes of *E.leyeensis* Deng, sp. nov. and *M.yaana* Deng, sp. nov. are shown in Table 3. With asymmetric nucleotide composition (*E.leyeensis* Deng, sp. nov.: 42.4% A, 30.2% T, 17.7% C, and 9.8% G; *M.yaana* Deng, sp. nov.: 44.3% A, 30.2% T, 16.5% C, and 9.3% G) and A+T-biased (*E.leyeensis* Deng, sp. nov.: 72.6%; *M.yaana* Deng, sp. nov.: 74.2%). This nucleotide composition pattern is consistent with other species of Tetrigidae (Xiao et al 2012a; Zhang et al 2021). The AT skews of *E.leyeensis* Deng, sp. nov. and *M.yaana* Deng, sp. nov. are 0.169 and 0.194, respectively, and the CG skews are -0.287 and -0.276, respectively. The AT-skew is positive, and the CG skew is negative. This shows that the content of bases C is higher than that of G, and A is higher than T in the whole (Table 3).

**Table 3.** Nucleotide composition of mitochondrial genome of *E.leyeensis* Deng, sp. nov. and *M.yaana* Deng, sp. nov.

Genes or partitions	A (%)	T (%)	G (%)	C (%)	A+T (%)	AT-skew	GC-skew
Whole genome	42.40/44.30	30.20/29.90	9.80/9.30	17.70/16.50	72.60/74.20	0.169/0.194	-0.287/-0.276
PCGs	31.30/34.60	39.80/38.00	13.70/12.30	15.20/.	71.10/72.60	-0.120/-0.047	-0.054/-0.106
PCGs-1^st^	34.30/37.90	34.40/32.60	17.90/16.30	13.00/13.30	68.70/70.50	0/0.075	0.155/0.100
PCGs-2^nd^	20.60/23.00	45.70/43.50	14.60/14.50	19.40/19.00	66.30/66.50	-0.379/-0.308	-0.149/-0.134
PCGs-3^rd^	39.00/42.90	39.40/37.90	8.50/6.00	13.00/13.20	78.40/80.80	-0.006/-0.061	-0.208/-0.372
tRNA	36.70/39.50	37.20/35.10	14.70/14.00	11.40/.	73.90/74.60	-0.008/0.059	0.130/0.104
rRNA	28.90/48.40	45.20/27.70	17.10/8.00	8.80/15.9	74.10/76.10	-0.220/0.272	0.319/-0.332
CR	49.74/51.09	30.97/32.67	6.49/6.62	12.80/9.62	80.70/83.80	0.233/0.220	-0.327/-0.185

Note: AT-skew = (A – T) / (A + T); GC-skew = (G – C) / (G + C).

#### Protein-coding genes and codon usage

The mitogenomes of both *E.leyeensis* Deng, sp. nov. and *M.yaana* Deng, sp. nov. contain 13 PCGs, with *nad5* being the longest and *atp8* being the shortest. The total length of the 13 PCGs in *E.leyeensis* Deng, sp. nov. and *M.yaana* Deng, sp. nov. are 11,098 bp and 11,116bp, respectively, approximately accounting for 70.18% and 67.87% of the whole mitogenome, respectively (Table 2). Nine of the 13 PCGs are encoded on the J-strand (*cox1*, *cox2*, *cox3*, *cytb*, *nad2*, *nad3*, *nad6*, *atp6*, *atp8*), and the other four (*nad1*, *nad4*, *nad4l*, *nad5*) are located on the N-strand (Table 2). ATN is the initiation codon of *E.leyeensis* Deng, sp. nov. and *M.yaana* Deng, sp. nov. (Table 2). Initiation codons for *nad2*, *nad6*, *nad4*, and *atp8* varied in two new species: *E.leyeensis* Deng, sp. nov. (ATG, ATA, ATT, ATA) and *M.yaana* Deng, sp. nov. (ATT, ATG, ATA, ATG). Both possess three types of stop codons: TAA (*cox1*, *cox2*, *cox3*, *nad1*, *nad2*, *nad4l*, *nad6*, *atp8*, *cytb*), TAG (*nad3*, *nad4*), and T- (*nad5*).

**Figure 2.**
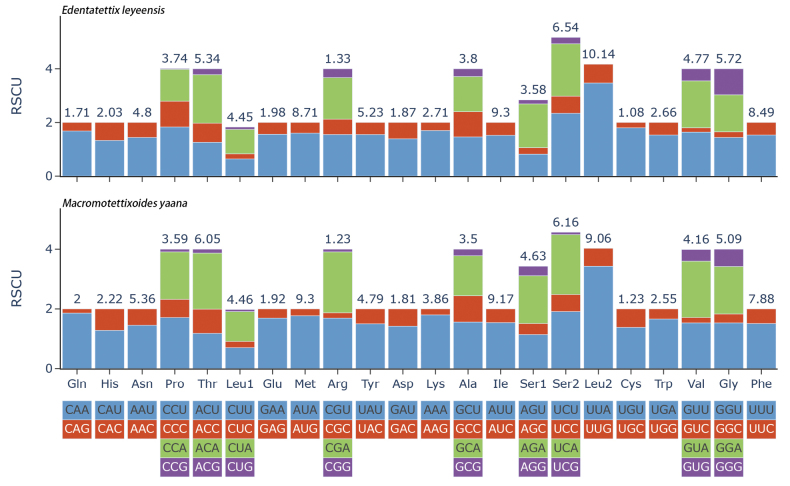
Relative synonymous codon usage (RSCU) in the mitogenomes of *E.leyeensis* Deng, sp. nov. and *M.yaana* Deng, sp. nov.

The relative synonymous codon usage (RSCU) values of the mitogenome are summarized (Fig. 2). The codon distribution analysis shows that the two codons UUA (Leu2) and UCU (Ser2) are the most frequently used in *E.leyeensis* Deng, sp. nov. The codons of UUA (Leu2) and UCA (Ser2) in *M.yaana* Deng, sp. nov. are the most frequently used. The frequency of the codons ending with A/U is much higher than with G/C, suggesting that the AU composition at the third position of codons has a positive influence on the nucleotide AT (or AU) bias of the PCGs.

#### Transfer and ribosomal RNA genes, and the A+T-rich region

**Figure 3.**
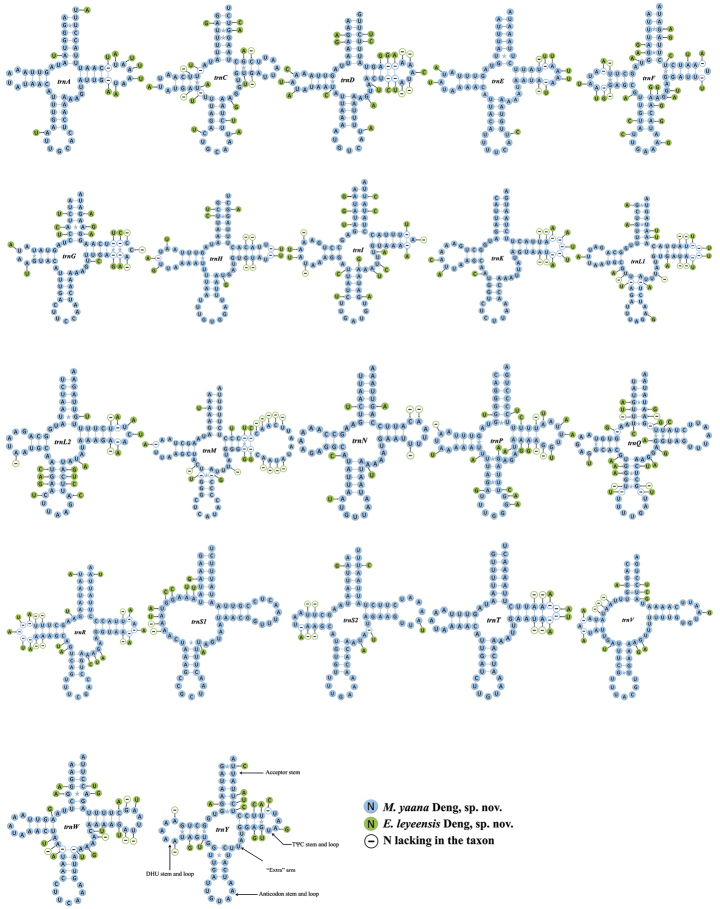
The secondary structures for 22 tRNA genes of *E.leyeensis* Deng, sp. nov. and *M.yaana* Deng, sp. nov. Watson-Crick base pairings and mismatches are represented by dashes (—) and stars, respectively.

The lengths of the 22 tRNA genes in *E.leyeensis* Deng, sp. nov. and *M.yaana* Deng, sp. nov. are 1418 bp and 1435 bp, respectively, with size ranges of 62 to 69 bp for *E.leyeensis* Deng, sp. nov. and 60 to 72 bp for *M.yaana* Deng, sp. nov. Two rRNA genes (*rrnL* and *rrnS*), separated by *trnV*, are located between *trnL1* and A+T-rich region. The total AT-skew of *E.leyeensis* Deng, sp. nov. and *M.yaana* Deng, sp. nov. in rRNA are -0.220 and 0.272, respectively. The total GC-skew of *E.leyeensis* Deng, sp. nov. and *M.yaana* Deng, sp. nov. in rRNA are 0.319 and -0.332, respectively. All tRNA genes could be folded into the typical clover-leaf structure, except *trnS1* and *trnV*, which lacked the dihydrouridine (DHU) arm (Fig. 3).

#### Phylogenetic analysis

**Figure 4.**
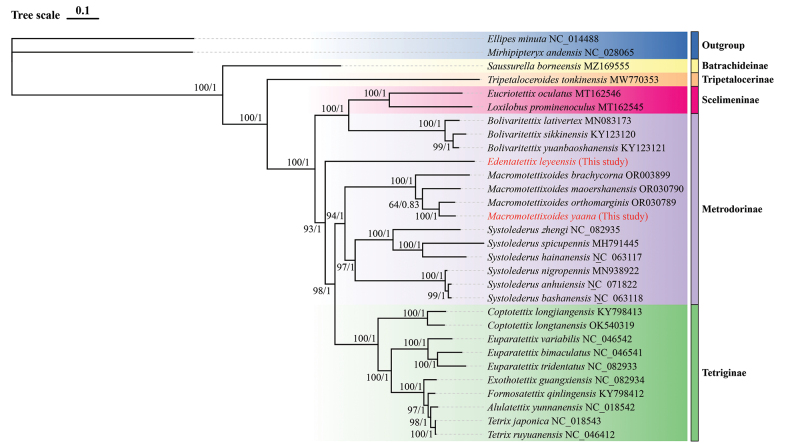
Phylogenetic tree obtained from ML and BI analysis based on 13 PCGs.

We constructed maximum likelihood (ML) and Bayesian inference (BI) trees based on the sequences of 13 PCGs from the mitochondrial genomes of 28 species from five subfamilies (Tetriginae, Metrodorinae, Scelimeninae, Tripetalocerinae, and Batrachideinae) of Tetrigidae and two outgroup species with the best partition schemes (Table 1). All phylogenetic analyses used the same data matrices, yet different methods yielded the same topology. Similar topologies were obtained from BI and ML trees (Fig. 4).

In this study, ML and BI trees had the same topological structures, and both phylogenies revealed the non-monophyletic relationships among species of the subfamily Metrodorinae. Tetrigidae was retrieved as monophyletic with strong support (posterior probability, PP = 1). However, only one species’ datum was available for Batrachideinae, and Tripetalocerina, making it impossible to determine their monophyly.

The results of phylogeny analysis conducted by ML and BI methods are as follows: (Batrachideinae + (Tripetalocerinae + ((Scelimeninae + Metrodorinae) + (Metrodorinae + (Metrodorinae + Tetriginae))))). *Saussurellaborneensis* Hancock, 1912 (Batrachideinae) split off earliest from the other taxa, suggesting that it is the earliest species within Tetrigidae. The subfamily Tetriginae represented the most evolutionarily advanced group within Tetrigidae, while the subfamily Metrodorinae occupied an intermediate position. These findings are consistent with previous studies (Chen 2005; Lin et al. 2015; Wang 2022). Although *E.leyeensis* Deng, sp. nov. bears a striking morphological similarity to species of *Macromotettixoides*, both belonging to wingless pygmy grasshoppers of the Metrodorinae, phylogenetic evidence does not justify classifying *E.leyeensis* within *Macromotettixoides*. Instead, it constitutes a separate new genus, namely *Edentatettix* Deng, gen. nov. Furthermore, another new species *M.yaana* Deng, sp. nov. forms a sister-group relationship with *Macromotettixoidesorthomargina* Wei & Deng, 2023, which is strongly supported (PP = 1), confirming that *M.yaana* Deng, sp. nov. belongs to *Macromotettixoides*.

#### Genetic distances

**Figure 5.**
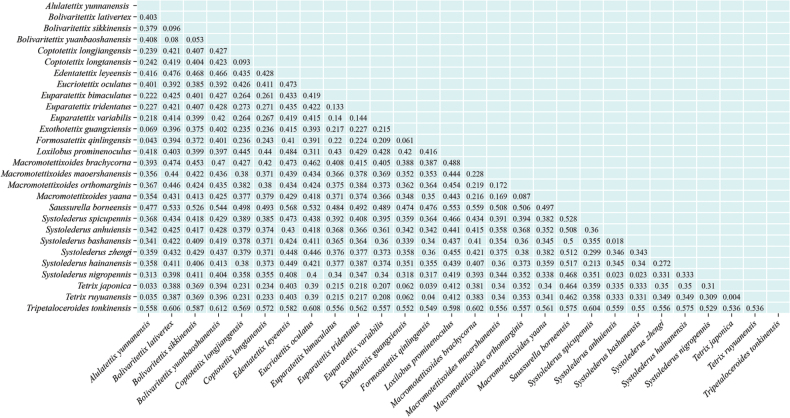
Pairwise genetic distances of mitochondrial DNA for 28 species of Tetrigidae based on 13PCGs.

In Tetrigidae, the genetic distances between different species pairs varied significantly, with a range from 0.018 to 0.612 (Fig. 5). In this study, the intraspecies genetic distance of *Tetrixjaponica* (= *Tetrixruyuanensis* Liang, 1998, syn. nov.) (Long et al. 2023) was 0.004. The genetic distances of the new species *M.yaana* Deng, sp. nov. to the three species of *Macromotettixoides* ranged from 0.083 to 0.200, which were greater than the interspecific distance (0.004), confirming that *M.yaana* Deng, sp. nov. is a separate species. Furthermore, the genetic distances between *E.leyeensis* Deng, sp. nov. and the other 27 species ranged from 0.403 to 0.582, which is significantly greater than the intergeneric genetic distances observed in *Systolederus* (0.018–0.360), *Bolivaritettix* (0.053–0.096), *Euparatettix* (0.133–0.227), and *Macromotettixoides* (0.087–0.228). Therefore, despite morphological similarities to species like *Macromotettixoides*, the genetic distances indicate that *E.leyeensis* Deng, sp. nov. warrants classification in a new genus.

#### Divergence time analysis

**Figure 6.**
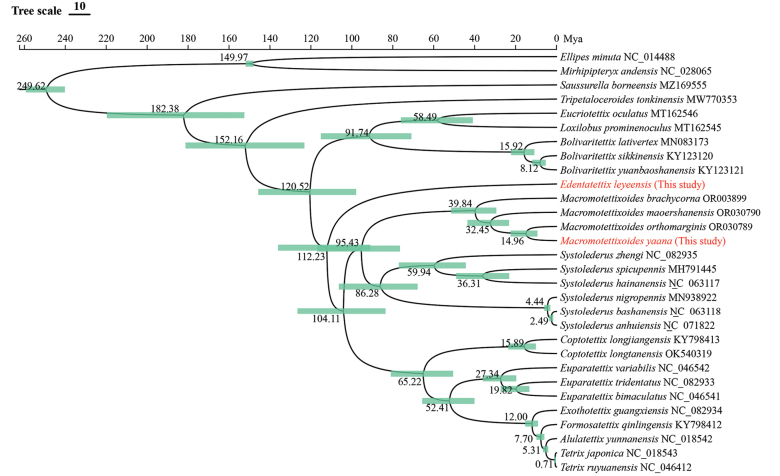
Dated phylogenetic tree of Tetrigidae based on the PCGs dataset. A time scale is provided. Mya refers to one million years ago.

Divergence time analysis shows that Metrodorinae appeared around 120.52 Mya (Fig. 6). This analysis also identifies considerable variability in the timings of differentiation across diverse genera within this subfamily. For example, the new genus *Edentatettix* Deng, gen. nov. can be traced back to approximately 112.23 Mya, which is close to the estimated split time of Metrodorinae. The early divergence of this genus may indicate distinct adaptive shifts in its evolutionary history, leading to the development of unique ecological niches and morphological characteristics. Additionally, *Tetrixjaponica*, with a divergence timescale of approximately 0.71 Mya, represents one of the most recently evolved species in Tetrigidae. It also exhibits the broadest global distribution among its relatives, reflecting its remarkable adaptability and ecological success.

### ﻿Taxonomy of the subfamily Metrodorinae Bolívar, 1887

#### ﻿Key to the Chinese genera of Metrodorinae Bolívar, 1887

Modified from Lu and Deng (2021), Metrodorinae currently includes 16 genera in China (including the new genus *Edentatettix* Deng, gen. nov.).

**Table:** 

1	Fastigium of the vertex distinctly projecting before anterior margin of compound eyes	**2**
–	Fastigium of the vertex not or slightly projecting before anterior margin of compound eyes	**4**
2	Fastigium of the vertex calyptriform protruding before anterior margin of compound eyes	***Calyptraeus* Wang, 2001**
–	Fastigium of the vertex angular projecting or square projecting	**3**
3	Fastigium of the vertex angular projecting; antennal grooves inserted between inferior margin of compound eyes	***Rhopalotettix* Hancock, 1910**
–	Fastigium of the vertex square projecting; antennal grooves inserted at lowest third of compound eyes height	***Miriatroides* Zheng & Jiang, 2002**
4	Saw-like teeth of female ovipositor absent or degenerate	***Edentatettix* Deng, gen. nov.**
–	Saw-like teeth of female ovipositor present	**5**
5	With a distinct obtuse projection under each lateral carina of prozona; pronotum platy, in dorsal view, dorsum of pronotum with irregular concavities	***Concavetettix* Deng, 2021**
–	Without obtuse projection under each lateral carina of prozona; pronotum tectiform	**6**
6	Vertex narrow, still narrower towards front, eyes drawn to each other in front and elevated	***Systolederus* Bolívar, 1887**
–	Vertex not as above	**7**
7	Posterior margins of each lateral lobes of pronotum only with ventral sinus	***Macromotettixoides* Zheng, Wei & Jiang, 2005**
–	Posterior margins of each lateral lobe of pronotum with ventral sinus and tegminal (upper) sinus	**8**
8	Humeral apex ridge and lower margin of pronotum connected in the middle or behind middle of lower margin of pronotum	***Macromotettix* Günther, 1939**
–	Humeral apex ridge and lower margin of pronotum connected before middle of lower margin of pronotum	**9**
9	Tegmina present and hind wings absent	***Paramphinotus* Zheng, 2004**
–	Tegmina and hind wings present	**10**
10	Ventral margins of fore femora and middle femora with two big teeth	***Orthotettixoides* Zheng, 1998**
–	Ventral margins of fore femora and middle femora without big teeth	**11**
11	Head and eyes not exserted above pronotal surface	**12**
–	Head and eyes distinctly exserted above pronotal surface	**13**
12	Anterior part of pronotum strongly widened, arched and uplifted	***Hyboella* Hancock, 1915**
–	Anterior part of pronotum normal and strongly widened	***Bolivaritettix* Günther, 1939**
13	Antennal grooves inserted far below inferior margin of compound eyes	**14**
–	Antennal grooves inserted at lowest third of compound eye height or between inferior margin of compound eyes	**15**
14	The vertex horn is distinctly raised above the dorsal margin of eyes and the vertex is deeply depressed between eyes in frontal view; pronotum between the shoulders is not elevated to an obtuse gibbosity	***Xistra* Bolívar, 1887**
–	In frontal view, the vertex horn slightly raised above the dorsal margin of eyes (or not) and the vertex is slightly depressed (or not depressed) between eyes; pronotum between the shoulders generally strongly elevated to an obtuse gibbosity	***Xistrella* Bolívar, 1909**
15	Antennal grooves inserted at lowest third of compound eye height; median carina of pronotum with a series of projections	***Cotysoides* Zheng & Jiang, 2000**
–	Antennal grooves inserted between inferior margin of compound eyes; median carina of pronotum generally straight or undulated	***Mazarredia* Bolívar, 1887**

### ﻿Descriptions

#### ﻿Genus Edentatettix

Taxon classificationAnimaliaOrthopteraTetrigidae

Deng gen. nov.

1770533B-3E00-596D-990F-EE51FA71EB1D

https://zoobank.org/A21EDA29-0040-4AF1-B899-87A4DB4D19DF

##### Type species.

*Edentatettixleyeensis* Deng, sp. nov., here designated.

##### Diagnosis.

The new genus can be easily distinguished from other genera of Metrodorinae by the saw-like teeth of the female ovipositor absent (Fig. 8G). *Edentatettix* is allied to *Macromotettixoides* Zheng, Wei & Jiang, 2005, but differs as follows: head and eyes slightly exserted above pronotal surface (not exserted in *Macromotettixoides*), dorsal surface of pronotum low and flat and tectiform is not obvious (distinctly tectiform in *Macromotettixoides*), saw-like teeth of female ovipositor absent (present in *Macromotettixoides*). *Edentatettix* is also similar to *Concavetettix* Deng, 2021 but differs from the latter by the obtuse projection under each lateral carina of the prozona absent (present in *Concavetettix*), dorsum of pronotum flat and slightly depressed in the middle part between the shoulders (dorsum of pronotum with irregular concavities in *Concavetettix*), saw-like teeth of female ovipositor absent (present in *Concavetettix*).

##### Description.

***General characters and coloration.*** Size small, brachypronotal. Coloration uniformly brown, antennae and face dark brown, middle of the dorsal surface of pronotum with a dark spot. Body surface is interspersed with sparse carinae and notches.

***Head.*** Head and eyes slightly exserted above pronotal surface. Fastigium of vertex short; in dorsal view, width of vertex between eyes 1.5–1.6× width of compound eye. In lateral view, frontal ridge and vertex forming a rounded-angle shape and slightly projected inferior margin of the compound eye, frontal costa distinctly concave between lateral ocelli. In frontal view, frontal costa bifurcated above lateral ocelli, the bifurcation of the frontal costa in the middle of the compound eye height; width of longitudinal furrow of frontal ridge narrower than antennal groove diameter. Antennae short, filiform, antennal grooves inserted below inferior margin of compound eyes. Eyes globose, lateral (paired) ocelli located lowest third of compound eye height.

***Thorax.*** Dorsal surface of pronotum low and flat and tectiform is not obvious; pronotal surface interspersed with sparse carinae and notches between shoulders and behind the middle, slightly depressed in the middle part between the shoulders. Pronotum with truncate anterior margin, median carina entire and nearly straight in profile; lateral carinae of prozona parallel; humeral angle obtuse; with interhumeral carina; hind pronotal process narrow, nearly reaching apex of hind femur and its apex narrowly rounded. Posterior angles of lateral lobes produced outwards, end of posterior angles truncate, posterior margins of lateral lobes of pronotum only with ventral sinus. Tegmina and hind wings invisible.

***Legs.*** Fore and middle femora slightly compressed, margins finely serrated and carinate and ventral margins slightly undulated. Hind femora robust and short, 2.8× as long as wide; with carinated. Length of first segment of posterior tarsi equal to third.

***Abdomen.*** Female ovipositor narrow and long, dorsal margins of upper valvulae and ventral margins of lower valvulae without saw-like tooth or saw-like teeth indistinct (Fig. 8G).

##### Etymology.

The generic epithet is derived from *edent*, referring to the absent saw-like teeth of female ovipositor (Fig. 8G).

#### ﻿ Edentatettix leyeensis

Taxon classificationAnimaliaOrthopteraTetrigidae

Deng sp. nov.

1B0EBF7D-8A3E-54C9-B45E-E4D87F64A277

https://zoobank.org/8DCD114B-F272-41D3-9C83-0426CD226421

Figs 7 , 8 , 9

##### Type material.

***Holotype*** • ♀, China, Guangxi Prov., Leye county (Wutaishan Forest Park), 24°51'11"N, 106°32'17"E, 1200 m alt., 23 August 2021, collected by Wei-An Deng, CLSGNU. ***Paratypes*** • 2♂, 5♀, same data, CLSGNU • 3♂, 6♀, same data, 18 August 2022, collected by Wei-An Deng and Yue-Mei Li, CLSGNU.

##### Diagnosis.

As there is only one species in the genus, see the generic diagnosis.

##### Description.

**Female.** Small size, short, body surface interspersed with sparse carinae and notches.

***Head*.** Head and eyes slightly exserted above pronotal surface. Fastigium of vertex short; in dorsal view, width of vertex between eyes 1.5–1.6× width of compound eye; anterior margin of fastigium arched and not surpassing anterior margin of eye; median carina visible; lateral margins turned backward; vertex uneven with paired fossulae. In lateral view, frontal ridge and vertex forming a rounded-angle shape and slightly projected inferior margin of the compound eye, frontal costa distinctly concave between lateral ocelli, protruding anteriorly and broadly rounded between antennal grooves. In frontal view, frontal costa bifurcated above lateral ocelli, the bifurcation of the frontal costa in the middle of the compound eye height; longitudinal furrow widely divergent between antennae, width of longitudinal furrow of frontal ridge narrower than antennal groove diameter. Antennae short, filiform, antennal grooves inserted below inferior margin of compound eyes, 15-segmented; the 10^th^ and 11^th^ segment are the longest, ~ 7.0–8.0× longer than its width. Eyes globose, lateral (paired) ocelli located lowest third of compound eye height.

***Thorax*.** Brachypronotal. Dorsal surface of pronotum low and flat and tectiform is not obvious; pronotal surface interspersed with sparse carinae and notches between shoulders and behind the middle, slightly depressed in the middle part between the shoulders. Pronotum with truncate anterior margin, median carina entire and nearly straight in profile; lateral carinae of prozona parallel; humeral angle obtuse; with interhumeral carina; hind pronotal process narrow, nearly reaching apex of hind femur and its apex narrowly rounded. Lower margin of hind process nearly straight, lateral carinae of metazona slightly curved, width of infrascapular area is 0.7 mm. Posterior angles of lateral lobes produced outwards, end of posterior angles truncate, posterior margins of lateral lobes of pronotum only with ventral sinus. Tegmina and hind wings invisible.

***Legs*.** Fore and middle femora slightly compressed, margins finely serrated and carinate and ventral margins slightly undulated. Hind femora robust and short, 2.8× as long as wide; with carinated, dorsal margins smooth and ventral margins finely serrated; antegenicular denticles and genicular denticles acute. Outer and inner side of hind tibia with five or six spines. Length of first segment of posterior tarsi equal to third, three pulvilli of first segment of posterior tarsi: first small, second and third large; apices of all pulvilli obtuse.

**Figure 7.**
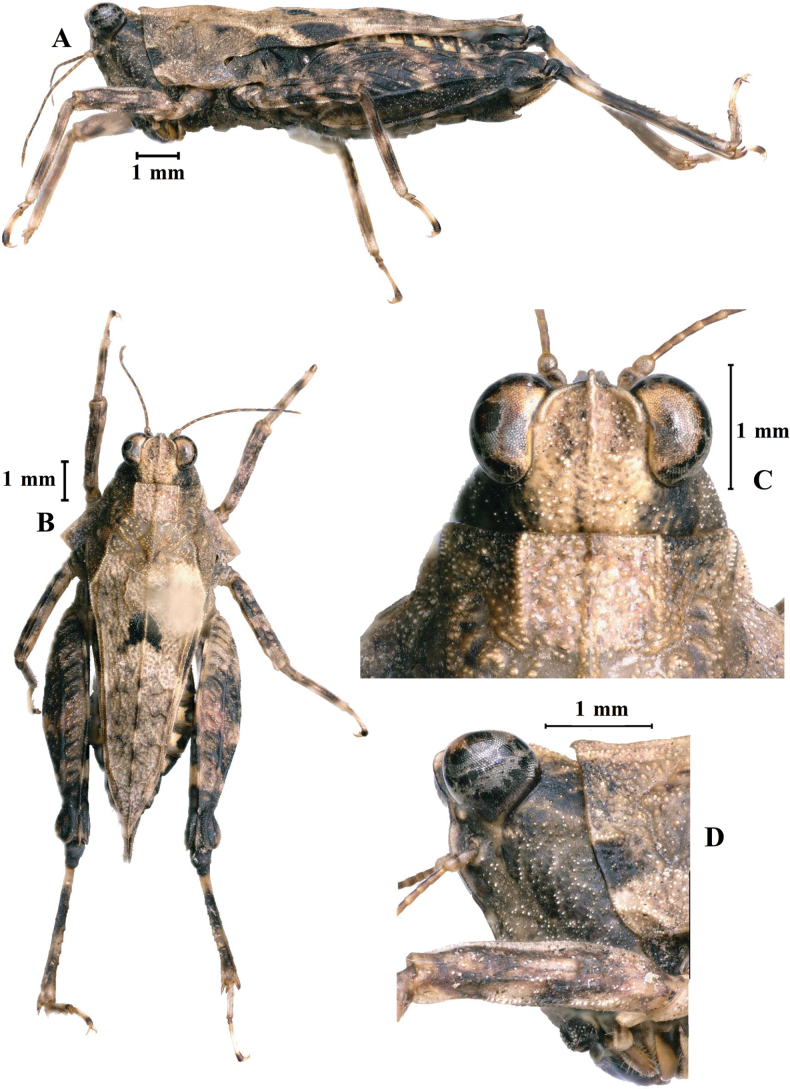
*E.leyeensis* Deng, sp. nov., holotype female **A** body, lateral view **B** the same, dorsal view **C** head and anterior part of pronotum, dorsal view **D** the same, lateral view.

**Figure 8.**
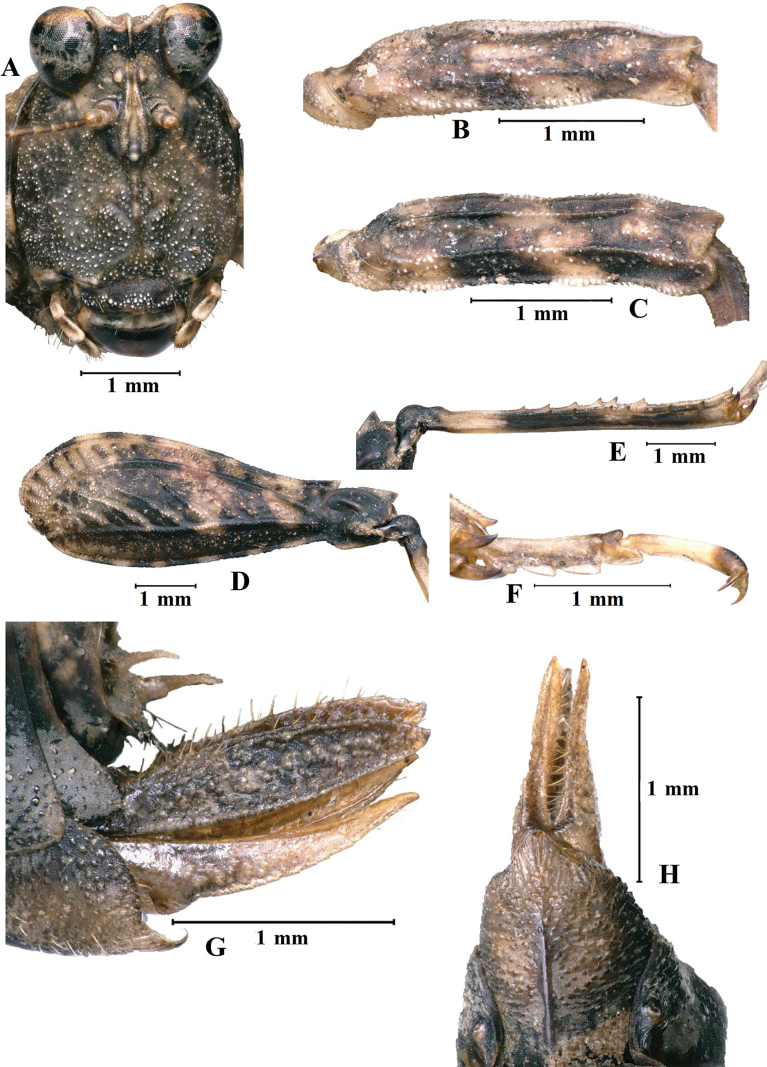
*E.leyeensis* Deng, sp. nov., holotype female **A** head, frontal view **B** left fore femur, lateral view **C** left mid femur, lateral view **D** left hind femur, lateral view **E** left hind tibia, lateral view **F** left posterior tarsus, lateral view **G** ovipositor of female, lateral view **H** subgenital plate of female, ventral view.

***Abdomen*.** Ovipositor narrow and long, length of upper valvulae 3.8× its width, dorsal margins of upper valvulae and ventral margins of lower valvulae without saw-like tooth or saw-like teeth indistinct (Fig. 8G). Length of subgenital plate slightly longer than its width, middle of posterior margin of subgenital plate triangular projecting.

**Figure 9.**
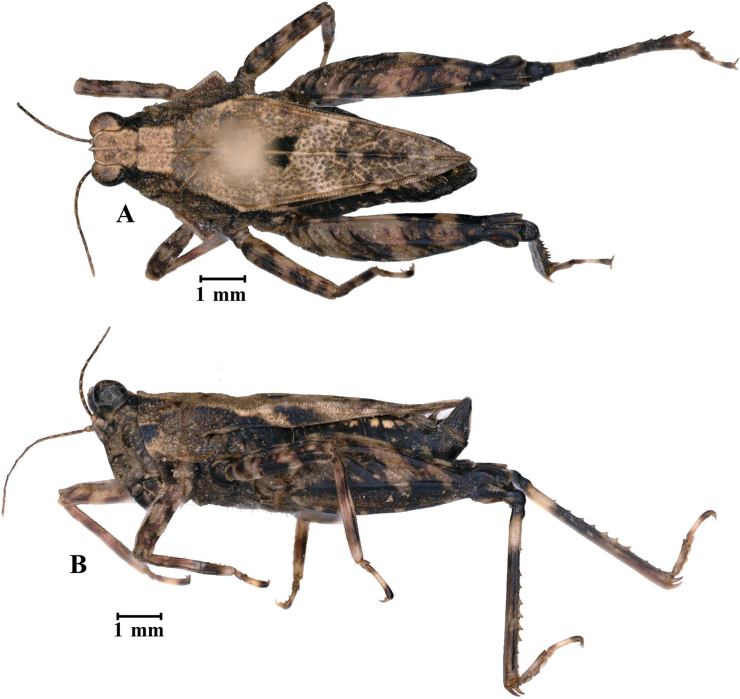
*Edentatettixleyeensis* Deng, sp. nov., paratype male **A** body in dorsal view **B** body in lateral view.

***Coloration*.** Body brown; antennae and face dark brown. Middle of dorsal surface of pronotum with a dark spot. Fore and middle femora and tibia brown, with two dark brown transverse spots. Hind femora dark brown, outer side with two pale stripes. Hind tibia black, with two pale rings in the middle.

**Male.** Similar to female, but smaller and narrower. Width of vertex between eyes 1.3–1.5× width of compound eye. Ventral margins of middle femora undulated. Subgenital plate short, cone-shaped, apex bifurcated.

##### Measurements (mm).

Length of body: ♂ 9.0–9.5, ♀ 11.5–12.0; length of pronotum: ♂ 8.0–8.5, ♀ 9.5–10.0; length of hind femur: ♂ 5.5–5.8, ♀ 6.5–7.0.

##### Etymology.

The specific name refers to the type locality: Leye, Guangxi, China; adjective.

##### Distribution.

P. R. China.

#### ﻿ Macromotettixoides yaana

Taxon classificationAnimaliaOrthopteraTetrigidae

Deng sp. nov.

FE869E6C-3F82-5D62-82D8-905DE603B10E

https://zoobank.org/63C2CA79-9127-44CB-A783-4E2F135833CB

Figs 11 , 12 , 13

##### Type material.

***Holotype*** • ♀, China, Sichuan Prov., Yaan City, Baoxing county, Longdong (Ganyanggou), 30°24′19″N, 102°35′45″E, 1400 m alt., 02 August 2016, collected by Wei-An Deng, CLSGNU. ***Paratypes*** • 8♂, 7♀, same data, CLSGNU.

##### Diagnosis.

This new species is similar to *Macromotettixoidesconvexa* Deng, 2020, from which it differs in that the pronotal surface is without a tuberculiform convex between shoulders (pronotal surface with a distinctly tuberculiform convexity between shoulders in *M.convexa*); lower margin of hind pronotal process straight (lower margin of hind pronotal process curved in *M.convexa*); median carina of pronotum undulated in profile (median carina of pronotum distinctly arch-like before shoulders and undulated behind shoulders in profile in *M.convexa*); lower outer carina of hind femora without projections (postmedian of lower outer carina of hind femora with two inconspicuous projections in *M.convexa*).

##### Description.

**Female.** Small size, short, body surface interspersed with coarse protuberances.

***Head*.** Head and eyes not exserted above pronotal surface. Fastigium of vertex short; in dorsal view, width of vertex between eyes 1.4–1.6× width of compound eye; anterior margin of fastigium slightly concave in the middle, slightly surpassing anterior margin of eye; median carina visible anteriorly; lateral margins turned backward; vertex uneven with paired fossulae. In lateral view, frontal ridge and vertex forming a rounded-angle shape, frontal costa distinctly concave between eyes, protruding anteriorly and broadly rounded between antennal grooves. In frontal view, frontal costa bifurcated above lateral ocelli, the bifurcation of the frontal costa in the middle of the compound eye height; longitudinal furrow widely divergent between antennae, width of longitudinal furrow of frontal ridge narrower than antennal groove diameter. Antennae short, filiform, antennal grooves inserted below inferior margin of compound eyes, 14-segmented, the 10^th^ and 11^th^ segment are the longest, ~ 2.5–3.0× longer than its width. Eyes globose, lateral (paired) ocelli located lowest third of compound eye height.

***Thorax*.** Brachypronotal. Pronotum with distinctly tectiform, pronotal surface interspersed with dense protuberances of variable sizes and notches. Pronotum with truncate anterior margin, median carina slightly lamellar and entire and undulated in profile; lateral carinae of prozona slightly lamellar and parallel; humeral angle obtuse; hind pronotal process narrow, nearly reaching apex of hind femur and its apex narrowly rounded. Lower margin of hind process straight, lateral carinae of metazona curved, width of infrascapular area is 0.9 mm. Posterior angles of lateral lobes slightly produced outwards, end of posterior angles truncate, posterior margins of lateral lobes of pronotum with distinctly ventral sinus and very weakly tegminal sinus. Tegmina and hind wings strongly reduced and covered by infrascapular area and invisible or slightly visible.

***Legs*.** Fore and middle femora slightly compressed, margins finely serrated and carinate and ventral margins with two inconspicuous projections and undulated. Hind femora robust and short, 2.8× as long as wide; with carinated and margins finely serrated; antegenicular denticles and genicular denticles acute. Outer side of hind tibia with 6–8 spines, inner side with six or seven spines. Length of first segment of posterior tarsi slightly longer than third, three pulvilli of first segment of posterior tarsi: first and second small and apices acute, third large and apex a right angle.

***Abdomen*.** Ovipositor narrow and long, length of upper valvulae 3.5× its width, upper and lower valvulae with slender saw-like teeth. Length of subgenital plate slightly longer than its width, middle of posterior margin of subgenital plate slightly triangular projecting.

***Coloration*.** Body dark brown or brown; antennae dark brown. Hind tibia dark brown, with two pale rings in the middle.

**Male.** Similar to female, but smaller and narrower. Width of vertex between eyes 1.4–1.5× width of compound eye. Ventral margins of fore and middle femora slightly undulated. Subgenital plate short, cone-shaped, apex bifurcated.

##### Measurements (mm).

Length of body: ♂ 6.5–7.0, ♀ 8.5–9.0; length of pronotum: ♂ 5.2–5.6, ♀ 7.0–7.5; length of hind femur: ♂ 4.0–4.5, ♀ 5.0–5.5.

##### Etymology.

The specific name refers to the type locality: Yaan, Jinxiu, Sichuan, China; adjective.

##### Distribution.

**Figure 10.**
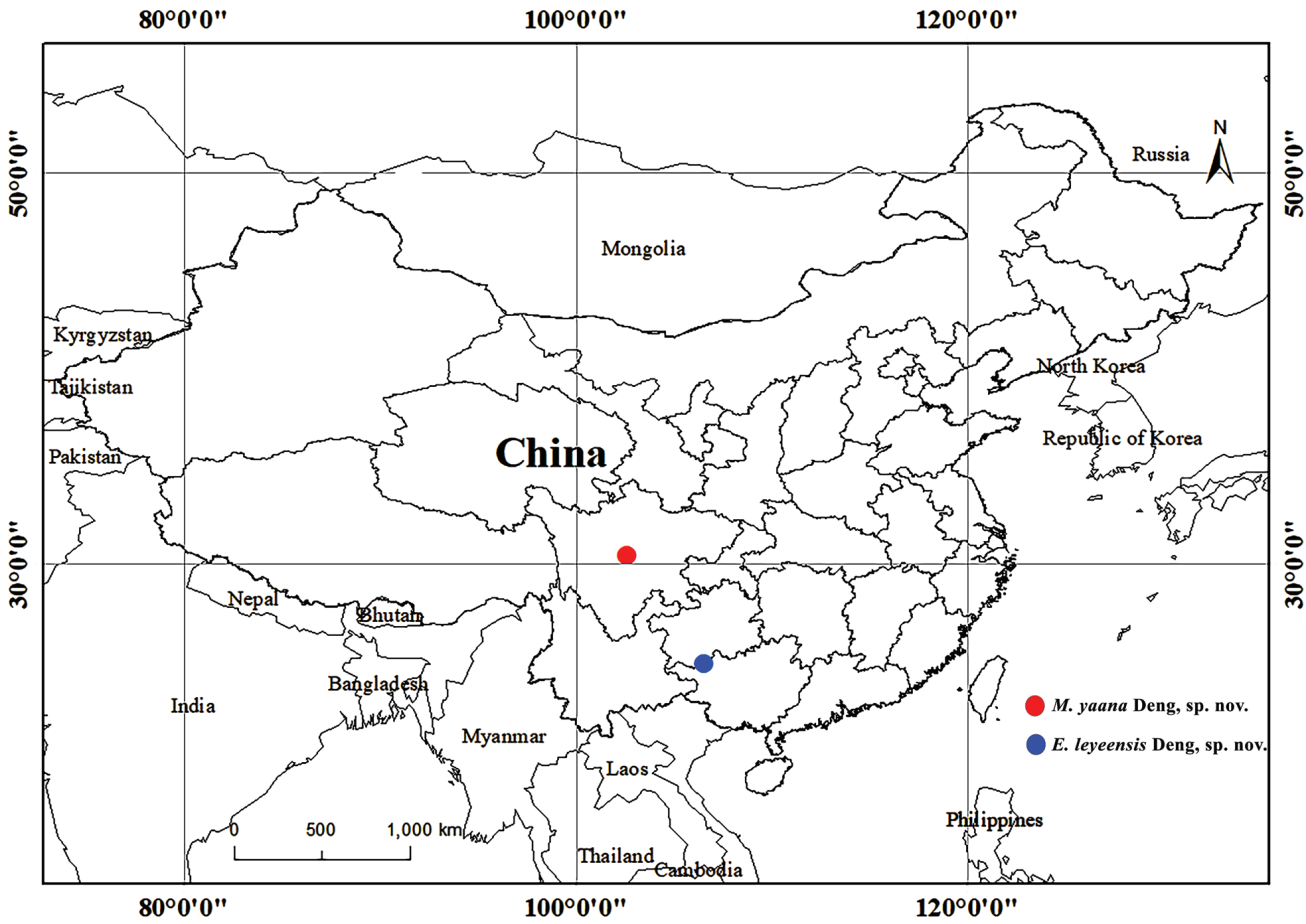
Distribution map of *E.leyeensis* Deng, sp. nov. and *M.yaana* Deng, sp. nov.

P. R. China: Sichuan (Fig. 10).

**Figure 11.**
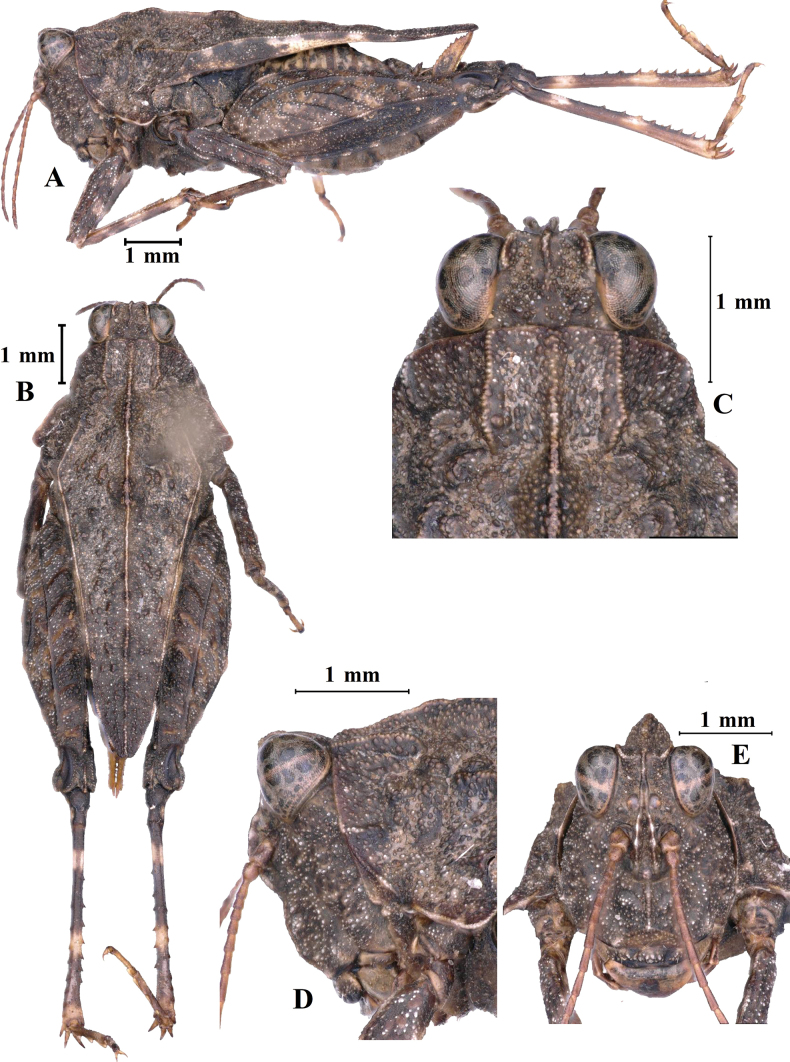
*Macromotettixoidesyaana* Deng, sp. nov., holotype female **A** body, lateral view **B** the same, dorsal view **C** head and anterior part of pronotum, dorsal view **D** the same, lateral view **E** head, frontal view.

**Figure 12.**
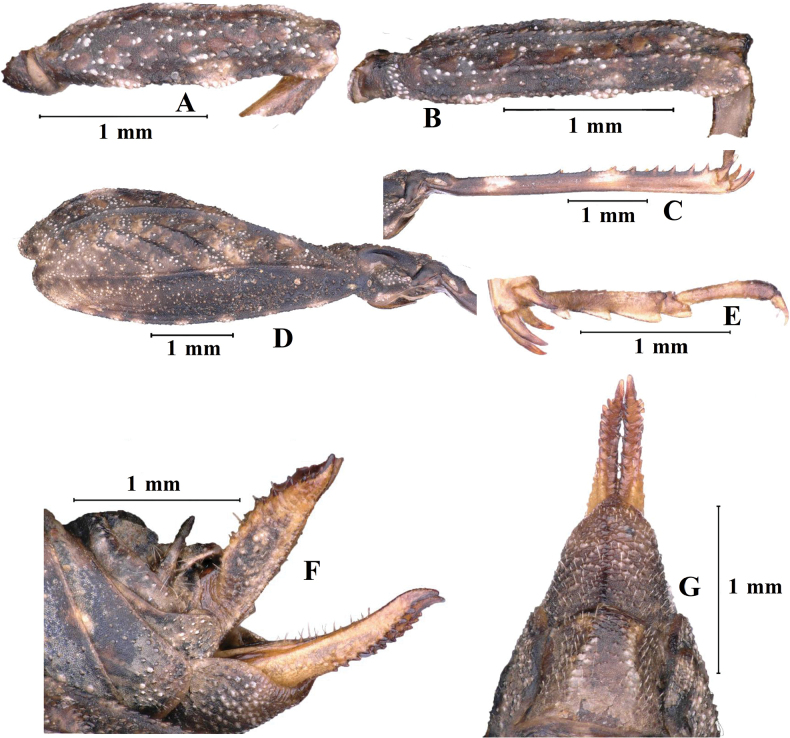
*Macromotettixoidesyaana* Deng, sp. nov., holotype female **A** left fore femur, lateral view **B** left mid femur, lateral view **C** left hind femur, lateral view **D** left hind tibia, lateral view **E** left posterior tarsus, lateral view **F** subgenital plate of female, lateral view **G** subgenital plate of female, ventral view.

**Figure 13.**
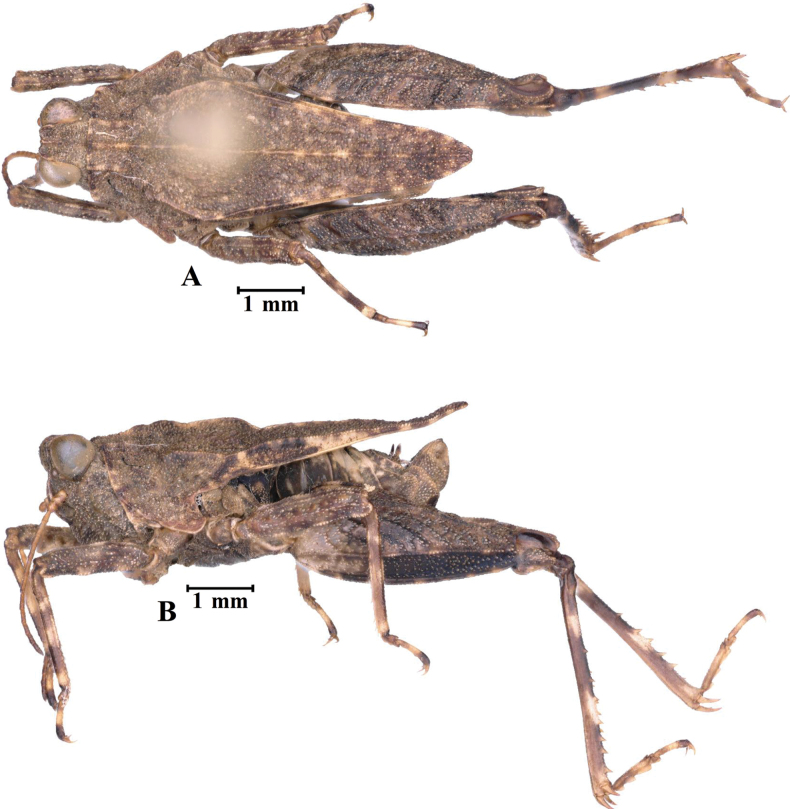
*Macromotettixoidesyaana* Deng, sp. nov., paratype male **A** body in dorsal view **B** body in lateral view.

### ﻿New synonyms

#### ﻿ Hainantettix strictivertex

Taxon classificationAnimaliaOrthopteraTetrigidae

Deng, 2020

F595D5C9-B73C-59E3-B18B-EB795B7DE718

Fig. 14

Hainantettix strictivertex Deng in Zhang, Zhao, Wu & Deng, 2020: 552 [description] (holotype – ♀, China: Hainan Prov., Qiongzhong, Limushan; paratypes – 4♀, China: Hainan Prov., Wuzhishan; in EMHU; examined).
Macromotettixoides angustivertex Zha & Peng in Peng, Shi, Ding & Zha, 2021: 48 [description] (holotype – ♀, China: Hainan Prov., Wuzhishan, in HNU, not examined).
Hainantettix angustivertex (Zha & Peng): Subedi 2022: 40; syn. nov.

##### Remarks.

*Macromotettixoidesangustivertex* Zha & Peng was described by Peng et al. (2021) and was later transferred to the genus *Hainantettix* by Subedi (2022). We examined the type specimen of *Hainantettixstrictivertex* Deng, 2020. Although we did not examine the type material of *M.angustivertex* from Hainan, according to the original description and photographs of the type specimens in Peng et al. (2021), we found that the two species share identical morphological structures, type locality, and coloration. These two taxa are conspecific and characterized by the vertex very strongly narrowed towards the front drawing the eyes together; antennal grooves inserted below inferior margin of compound eyes; rami strongly divergent, width of longitudinal furrow of frontal ridge is distinctly wider than antennal groove diameter.

**Figure 14.**
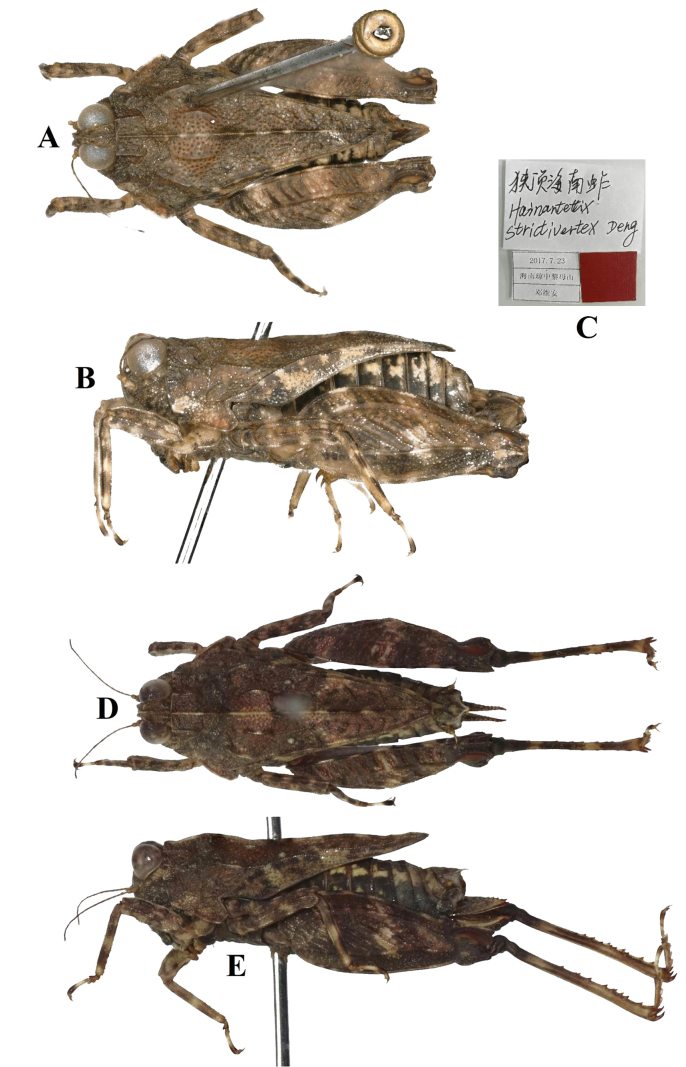
**A–C** Holotype of *Hainantettixstrictivertex* Deng, 2020 **A** body in dorsal view **B** body in lateral view **C** labels **D, E** holotype of *Macromotettixoidesangustivertex* Zha & Peng, 2021, syn. nov. **D** body in dorsal view **E** body in lateral view (photographs from Peng et al.).

#### ﻿ Macromotettixoides jiuwanshanensis

Taxon classificationAnimaliaOrthopteraTetrigidae

Zheng, Wei & Jiang, 2005

88DBC16A-CDC8-523B-B5D6-D51F6C9B42D4

Figs 15 , 16

Macromotettixoides jiuwanshanensis Zheng, Wei & Jiang, 2005: 366 [description] (holotype – ♀, China: Guangxi Prov., Luocheng (jiuwanshan), in IZSNU, examined). Zheng et al. 2005b: 176; Zheng et al. 2006: 604; Deng et al. 2007: 160; Zheng and Shi 2009: 572; Deng 2011: 544; Zheng 2013a: 242; Deng et al. 2014: 548; Deng et al. 2015:1 65; Deng 2016: 156; Zha et al. 2017: 16; Han et al. 2020: 563; Li et al. 2020a: 110; Fan et al. 2023: 128.
Hyboella badagongshanensis Zheng, 2013b: 11 (holotype – ♀, China: Hunan Prov., Sangzhi (Badagongshan), in IZSNU, examined); Deng 2016: 150; syn. nov.
Macromotettixoides badagongshanensis (Zheng, 2013b): Zha, Yu, Boonmee, Eungwanichayapant, Wen, 2017: 23; Han et al. 2020: 564; Li et al. 2020a: 112; Fan et al. 2023: 129; syn. nov.
Macromotettixoides wuyishana Zheng, 2013: 242[description] (holotype – ♀, China: Fujian Prov., Wuyishan, in IZSNU, examined); Deng 2016: 156; Zha et al. 2017: 15; Han et al. 2020: 563; Li et al. 2020a:1 11; Fan et al. 2023: 128; syn. nov.

##### Remarks.

*Hyboellabadagongshanensis* was described by Zheng (2013b) but was later transferred to the genus *Macromotettixoides* by Zha et al. (2017). We examined the type specimens of *M.jiuwanshanensis*, *M.badagongshanensis*, and *M.wuyishana* and found that the structures and coloration of the body are the same in these three taxa. Therefore, we consider *M.badagongshanensis*, and *M.wuyishana* as synonyms of *M.jiuwanshanensis*. These three taxa are conspecific and characterized by the width of the vertex between the eyes being 2.1–2.2× the width of the compound eye; anterior margin of fastigium arched and surpassing anterior margin of eye; the anterior margin of pronotum is slightly obtuse protruding; and the lower carinae of fore and mid femora is straight.

**Figure 15.**
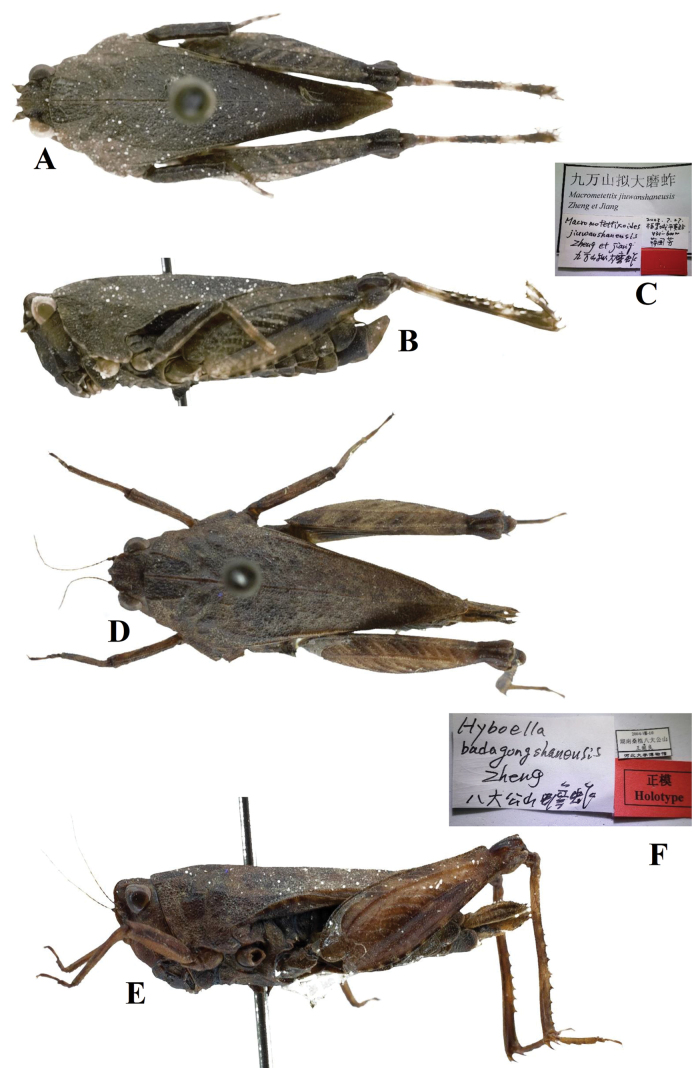
**A–C** Holotype of *Macromotettixoidesjiuwanshanensis* Zheng, Wei & Jiang, 2005 **A** body in dorsal view **B** body in lateral view **C** labels **D–F** holotype of *Hyboellabadagongshanensis* Zheng, 2013, syn. nov. **D** body in dorsal view **E** body in lateral view **F** labels.

**Figure 16.**
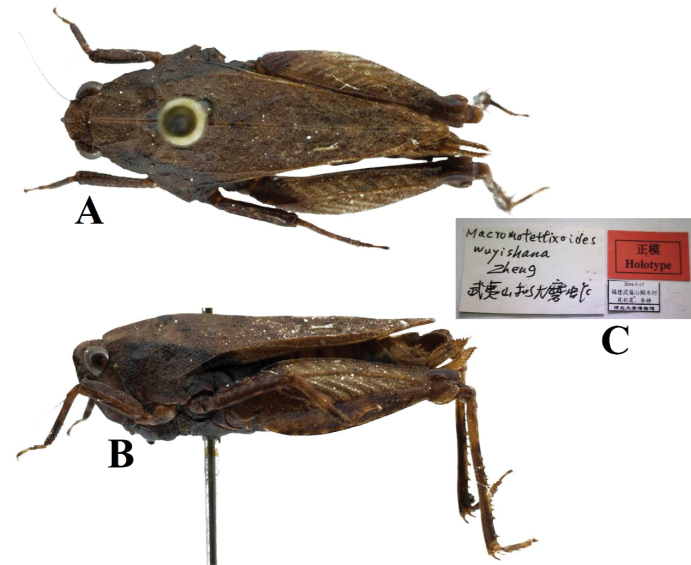
*Macromotettixoidesjiuwanshanensis* Zheng, Wei & Jiang, 2005. Holotype of *Macromotettixoideswuyishana* Zheng, 2013, syn. nov. **A** body in dorsal view **B** lateral view **C** body in labels.

## ﻿Discussion

**Figure 17.**
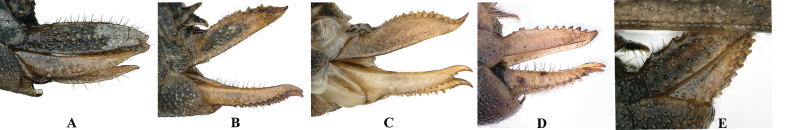
ovipositor of female, lateral view **A***E.leyeensis* Deng, sp. nov. **B***M.yaana* Deng, sp. nov. **C***M.orthomargina***D***M.brachycorna***E***M.maoershanensis*.

In this study, we describe two new pygmy grasshoppers of Metrodorinae found in China, *E.leyeensis* Deng, sp. nov. and *M.yaana* Deng, sp. nov. Through detailed morphological description and mitochondrial genome sequencing, we conducted a comprehensive analysis of the two new species. We have discovered something quite intriguing. Although *E.leyeensis* Deng, sp. nov. morphologically resembles the genus *Macromotettixoides*, its uniqueness lies in the absence of serrated teeth on the female ovipositor (Fig. 17), which is extremely rare among Tetrigidae. Based on morphological characteristics, phylogeny, and divergence time analysis, *E.leyeensis* Deng, sp. nov. is set apart in classification, leading us to classify it under the new genus *Edentatettix* Deng, gen. nov.

The existence of an ovipositor serves as a shared derived characteristic (synapomorphy) among Insecta (Barbosa and Fianco 2024). Across this expansive class, a diverse array of morphological and functional characteristics can be observed in the ovipositors among different orders, and the varied structures of ovipositors in Orthoptera are no exception, vividly mirroring their unique adaptations to diverse ecological niches (Austin and Browning 1981; Kluge 2016). For instance, the sword-like or spear-shaped ovipositors of katydids and crickets exemplify their high adaptability for laying eggs in plant tissues or soil, ensuring both egg safety and enhanced larval survival rates (Kluge 2016; Chen et al. 2021). Conversely, the ovipositors of locusts and pygmy grasshoppers (Tetrigidae) are geared more towards excavating functionality, with the former having a conical shape facilitating efficient soil digging (Kalogianni 1995; Zhao et al. 2022). The latter, while also adept at digging, may further enhance oviposition precision and efficiency by having fine teeth along the dorsal and ventral edges of their ovipositors (Kluge 2016). These varied oviposition strategies typify Orthopteran responses to environmental pressures, embodying a co-evolutionary mechanism between form and function.

The emergence of *E.leyeensis* Deng, sp. nov. with a toothless ovipositor provides not only morphological evidence for early Tetrigidae diversification but also insights into the ancient origins of complex reproductive structure transformations. The toothless ovipositor may represent a unique adaptive strategy, potentially associated with specific oviposition habitats or behaviors, meriting further investigation.

Importantly, we note for the first time that toothless ovipositors are an important taxonomic feature. This distinctive feature intimates the likelihood of diverse morphological variations in reproductive organs that may have emerged throughout the evolutionary journey of this group. Such variations are plausibly influenced by a multitude of factors, encompassing genetics, environmental conditions, and reproductive strategies (Hornig et al. 2018; Chen et al. 2021; Barbosa and Fianco 2024). Moreover, the timing of the emergence of this toothless characteristic (*E.leyeensis* Deng, sp. nov.), paralleling the divergence period of Tetrigidae, implies that it is an ancient character. This finding reinforces the hypothesis that Tetrigidae insects have undergone intricate morphological transformations, implicating their reproductive structures, over the course of their evolutionary progression.

Fossil records from the Early Miocene and Middle Eocene epochs affirm that the ovipositors of female Tetrigidae were adorned with saw-like teeth (Heads et al. 2014; Skejo et al. 2024), congruent with observations in contemporary species. The ancient divergence time of *E.leyeensis* Deng, sp. nov., given the constraints posed by the fossil record, accentuates that our current paleontological evidence likely constitutes merely a fraction of the extensive and diverse history of life. Some species, especially those inhabiting environments unfavorable to fossil preservation, may have irretrievably lost their historical footprint. Consequently, integrating insights from molecular biology, morphology, and paleontology becomes indispensable for elucidating the profound evolutionary mechanisms underlying global biodiversity.

## Supplementary Material

XML Treatment for Edentatettix

XML Treatment for Edentatettix leyeensis

XML Treatment for Macromotettixoides yaana

XML Treatment for Hainantettix strictivertex

XML Treatment for Macromotettixoides jiuwanshanensis

## ﻿Additional information

### Author ORCIDs

Yuemei Li https://orcid.org/0009-0001-7327-5334

Shixiong Leng https://orcid.org/0009-0002-3422-3862

Jiasong He https://orcid.org/0009-0002-7224-594X

Weian Deng https://orcid.org/0000-0002-8023-2498

Delong Guan https://orcid.org/0000-0001-6866-0943

### Data availability

All of the data that support the findings of this study are available in the main text.
